# Premenopausal Women With a Diagnosis of Endometriosis Have a Significantly Higher Prevalence of a Diagnosis or Symptoms Suggestive of Restless Leg Syndrome: A Prospective Cross-Sectional Questionnaire Study

**DOI:** 10.3389/fendo.2021.599306

**Published:** 2021-03-29

**Authors:** Nicola Tempest, Madeleine Boyers, Alice Carter, Steven Lane, Dharani K. Hapangama

**Affiliations:** ^1^ Department of Women’s and Children’s Health, Institute of Life Course and Medical Sciences, University of Liverpool, Member of Liverpool Health Partners, Liverpool, United Kingdom; ^2^ Liverpool Women’s Hospital NHS Foundation Trust, Member of Liverpool Health Partners, Liverpool, United Kingdom; ^3^ Department of Biostatistics, Institute of Life Course and Medical Sciences, University of Liverpool, Member of Liverpool Health Partners, University of Liverpool, United Kingdom

**Keywords:** endometriosis, restless leg syndrome, chronic disease, pelvic pain, depression, steroid hormones, multimorbidity

## Abstract

**Background:**

Endometriosis and restless leg syndrome (RLS) are both chronic conditions that can negatively affect a woman’s quality of life. A higher prevalence of RLS is seen in women and particularly in those who are pregnant, suggesting a possible ovarian hormonal influence. Endometriosis is a common (affecting 1 in 10 women) estrogen driven gynecological condition, and the prevalence of RLS in women with symptoms or a diagnosis of endometriosis is unknown.

**Methods:**

A prospective, cross-sectional, observational self-completed questionnaire study was distributed to 650 pre-menopausal women attending the gynecological department at Liverpool Women`s Hospital over a period of 4 months. 584 questionnaires were returned and 465 completed questionnaires were included in the final dataset. Data on RLS-associated (The International Restless Leg Syndrome Study Group rating scale) and endometriosis-associated (modified-British Society of Gynaecological Endoscopists pelvic pain questionnaire) symptoms were collected.

**Results:**

Women who reported a prior surgical diagnosis of endometriosis had a greater risk of having a prior formal diagnosis of RLS (OR 4.82, 95% CI 1.66,14.02) and suffering RLS symptoms (OR 2.13, 95% CI 1.34-3.39) compared with those without a diagnosis. When women with either a formal surgical diagnosis or symptoms associated with endometriosis were grouped together, they also have a significantly increased risk of having either a formal diagnosis or symptoms suggestive of RLS (OR 2.49, 95% CI 1.30, 3.64). In women suffering with endometriosis-associated symptoms, the cumulative endometriosis-associated symptom scores demonstrated a modest positive correlation with RLS severity scores (r=0.42 95% CI 0.25 to 0.57).

**Conclusions:**

This is the first study highlighting an association between the symptoms relevant to the two chronic conditions RLS and endometriosis, showing that women with a reported prior surgical diagnosis or symptoms suggestive of endometriosis have a significantly higher prevalence of a prior formal diagnosis or symptoms suggestive of RLS. This data will help in facilitating the discovery of novel therapeutic targets relevant to both conditions. The simultaneous treatment of these conditions could potentially lead to improvement in the overall quality of life for these women.

## Introduction

Endometriosis is a common, chronic, estrogen driven condition, occurring almost exclusively in women of reproductive age. It is defined as the growth of endometrium-like tissue, beyond the usual place, the uterine cavity (1). The prevalence among women of reproductive age is reported to be 10% while 25-50% of infertile women are reported to have a surgical diagnosis of endometriosis at laparoscopy (2, 3). The complex pathophysiology of ectopic growth of the endometrium is not fully understood (1). Laparoscopy is the gold standard diagnostic tool, with analgesics and hormonal contraceptive medications recommended as first line treatment (4, 5). At present, no curative treatments are available (5) and the existing evidence for disease progression is conflicting. Endometriosis is therefore a challenging condition to manage, with a significant cost to the suffering women, their families, the health service and society in general. More than half of the women with a diagnosis of endometriosis reported their symptoms to negatively impact on their quality of life (QoL) affecting their relationships and jobs, general physical and psychological health, and social functioning (6). Current literature further suggests that women with endometriosis have an increased prevalence of chronic pain syndromes including irritable bowel syndrome (IBS), fibromyalgia, painful bladder syndrome/interstitial cystitis and vulvodynia, which will add to their symptom burden (7–13). Therefore, improving the management of endometriosis associated chronic symptoms is a major unmet need in women’s health (6).

Restless Leg Syndrome (RLS) also known as Willis-Ekbom disease, is a sensory-motor disorder, affecting around 5-10% of the general population (14). The pathognomonic feature is the irresistible urge to move the legs due to an unpleasant non-painful sensory disturbance, described in a variety of ways for example as crawling, creeping and pulling (refer to (15, 16). The etiology of RLS, like endometriosis, is poorly understood. The National institute of clinical excellence (NICE) in the UK recommends conservative therapy for mild-moderate symptoms of RLS and pharmacotherapy for moderate-severe RLS with more frequent symptoms. However, effective symptom control remains difficult, with persistent symptoms often negatively impacting on a patients quality of life (17).

The relationship between endometriosis and female sex steroid hormones is well recognized (1). RLS is twice as common in women compared to men, and more common in pregnancy (18), indicating a possible hormonal basis (14). The relation of both conditions to other chronic pain syndromes and their possible hormonal influence led us to hypothesize that endometriosis and RLS may be linked. This study therefore, was conducted to assess the previously unknown prevalence of RLS associated symptoms among non-pregnant, premenopausal women and to ascertain if a possible prior surgical diagnosis or suffering with symptoms associated with endometriosis, will alter the prevalence of RLS symptoms.

## Materials and Methods

This questionnaire study was approved by the North of Scotland Research Ethics Committee 2 (LREC: 17/NS/0070).

### Questionnaire Development

The International Restless Leg Syndrome Study Group (IRLSSG) Severity Rating Scale (19) was identified as the most suitable validated, clinical tool (self-completed questionnaire) for assessing RLS associated symptoms, to indicate a possible diagnosis of RLS and severity of symptoms. Since there are currently no reliable self-administered diagnostic questionnaires for endometriosis, to identify the presence of endometriosis associated symptoms, two validated and widely used tools were considered, the Endometriosis Phenome (and Biobanking) Harmonisation Project (EPHect) Endometriosis Patient Questionnaire (EPQ) a 25-page document (20), alongside the shorter British Society for Gynecological Endoscopy (BSGE) pelvic pain questionnaire (21). The questionnaire was modified according to the feedback from a group of gynecological patients who volunteered to attend a focus group at the Liverpool Women’s hospital (LWH). The attendees (n=10) found EPHect EPQ to be too lengthy and reported that it would be unacceptable to them as completion before leaving the clinic will be tedious. Subsequently, to obtain a higher return of fully completed questionnaires, their preferred option, the modified BSGE pelvic pain questionnaire for endometriosis associated symptoms was selected for our study. IRLSSG and BSGE questionnaires were then merged to devise the final study questionnaire (**Supplementary Figure 1**), and its suitability and acceptability was confirmed in a preliminary pilot study, including 15 gynecological patients of reproductive age.

### Data Collection and Analysis

The questionnaire was distributed to 650 gynecological patients under the age of 50, attending the gynecology outpatient department (which included the general gynecology clinics, and specialist clinics such as endometriosis, colposcopy, urogynecology and fertility) at LWH from the 5th of October 2017 to the 11^th^ of January 2018.

Verbal and written (participant information sheet) information was imparted at the distribution of the questionnaire by the researchers. This detailed that consent is assumed on the voluntary return of the completed questionnaire, which did not contain any personal identifiable data. Questionnaires were considered eligible only when all questions were answered and the data provided by the responders fulfill the inclusion criteria of being non-pregnant females of 18-50 years of age. Previous studies have indicated an increased prevalence of RLS in pregnancy, thus pregnant women were excluded and since endometriosis almost exclusively affects women of reproductive age, the study only included women between 18-50 years of age.

The demographic data collected in the questionnaire included age, confirmation of non-pregnant status, use of hormonal and other medications, menstrual history, parity, smoking, alcohol intake and whether women had a prior surgical diagnosis of endometriosis/prior formal diagnosis of RLS. In the UK, a formal diagnosis of RLS is made by an appropriate specialist physician (for example, a sleep specialist or a neurologist) according to the standard guidance, fulfilling the criteria defined by the IRLSSG as recommended by NICE (17). The endometriosis associated symptoms questionnaires collected data on premenstrual, menstrual, and non-cyclical pelvic pain, dyspareunia, bowel/bladder associated pain, and back pain with a visual analogue score (VAS) indicating the severity of each pain symptom reported. We defined a score of ≥7 VAS for any of these pain symptoms to be indicative of the responder suffering with possible endometriosis-associated symptoms. Women were categorized to the group suffering with symptoms suggestive of RLS, when the participant responded “yes” to the question “Do you ever have the irresistible urge to move your legs due to an unpleasant sensation?”. The severity of RLS was calculated by the cumulative sum of 10 RLS VAS responses as previously described (22). Although endometriosis cannot be diagnosed efficiently with the analysis of symptoms, the symptoms of pelvic pain that are associated with endometriosis remain a major therapeutic challenge. Therefore, we attempted to assess the severity of these symptom perceived by the respondents of our study by calculating the cumulative sum of the 8 endometriosis associated pain symptom VAS responses.

### Sample Size Calculation

Our initial calculations suggested a minimum sample size of 200 would be sufficient to draw conclusions, yet due to the heterogeneous nature of the symptoms associated with endometriosis in particular, we aimed to collect approximately 400 responses. We distributed 650 questionnaires assuming over 60% return of fully completed responses from eligible patients within our study period.

### Statistical Analysis

The study population was divided into groups, considering those with a surgical diagnosis or symptoms associated with endometriosis and those without, the two main outcome measures included a prior diagnosis of RLS and presence of RLS associated symptoms (based upon the responses to the question ‘Do you ever have the irresistible urge to move your legs due to an unpleasant sensation?’). Those who responded ”yes” were classified as a case and those who responded ”no” a non-case. Initially summary data for the different groups were extracted and reported using standard summary statistics, mean and standard deviation for continuous data and counts and percentages for categorical data. Hypothesis test using independent ANOVA and Chi-squared tests were undertaken depending on the distribution of the data. The odds-ratio was used to quantify the risk of RLS when comparing those with confirmed diagnosis or reported symptoms suggesting endometriosis and those without symptoms. We investigated the association between continuous endometriosis scores and continuous RLS severity scores using Spearman correlation.

Statistical analysis was performed using SPSS 21.0 for Windows (SPSS Inc., Chicago, IL, USA).

## Results

**Figure 1:**
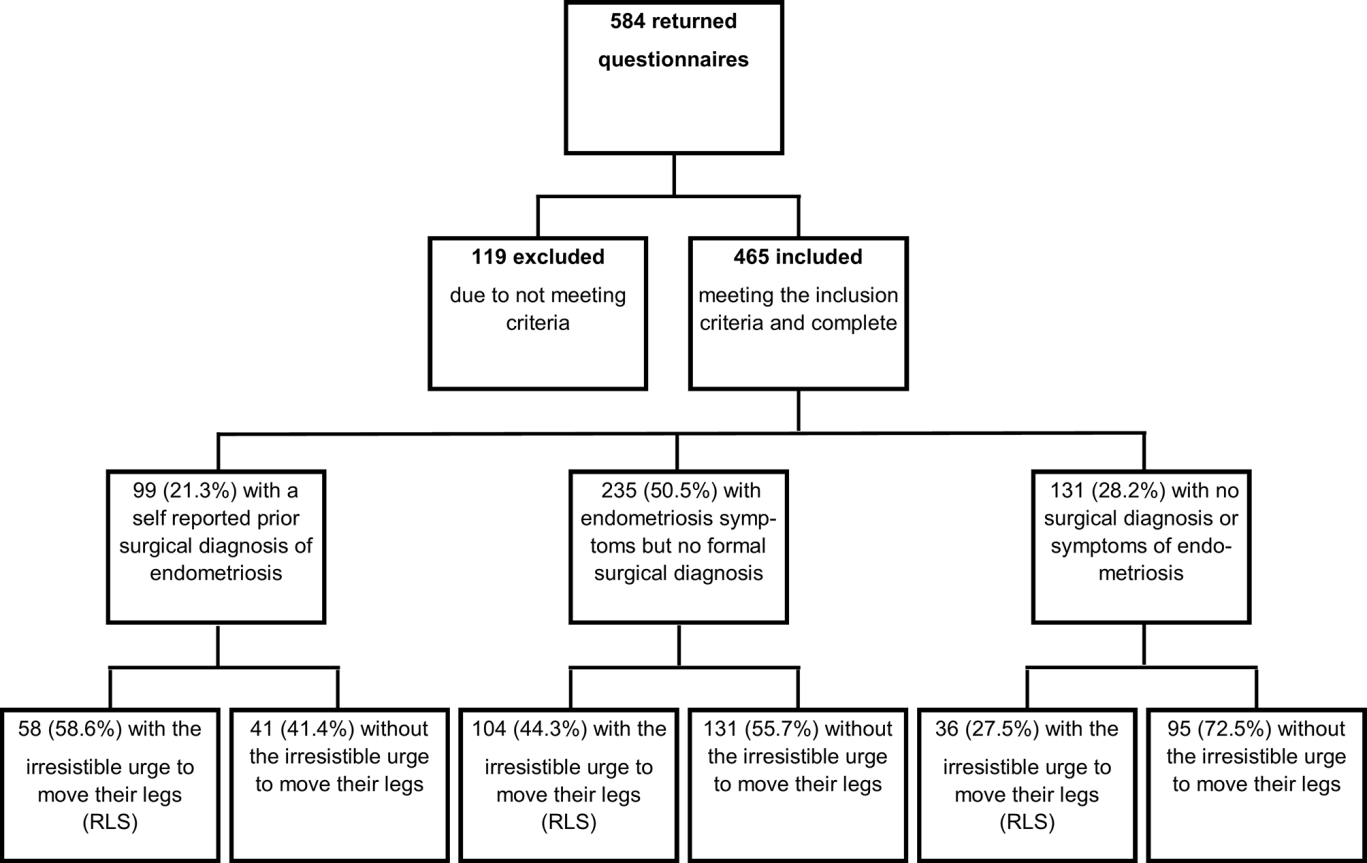
Flow chart documenting questionnaire responses.

The overall response rate for this study was high (90%, 584/650), 119 questionnaires were excluded due to incomplete responses or responders not meeting all pre-determined eligibility criteria. Therefore, the eligible number of questionnaires included in the final analysis was 465 (71.5%) (**Figure 1**).

Features of the patient population are detailed in **Table 1** (segregated by endometriosis status) and **Table 2** (separated by RLS status).

**Table 1:** Demographics separated by endometriosis status.

	Surgical endometriosis diagnosis	Endometriosis symptoms, no formal diagnosis	No diagnosis/symptoms of endometriosis	P value
	(n=99)	(n=235)	(n=131)	
Age in years, mean (SD)	35.6 (8)	32.4 (8.6)	33.2 (8.1)	<0.01^1^
Hormonal medication, n (%)	50 (50.5)	80 (34)	43 (32.8)	<0.01^2^
Smoker, n (%)	10/97 (10.3)	54/226 (23.9)	25/121 (20.7)	0.022
Alcohol consumption, n (%)	43/97 (44.3)	132/224 (58.9)	70/130 (53.8)	0.052
Parous women, n (%)	53/96 (55.2)	113/226 (50)	63/119 (52.9)	0.672
Anti-depressant use, n (%)	20 (20.2)	43 (18.3)	13 (9.9)	0.062

^1^ANOVA; ^2^Chi-squared

**Table 2:** Demographics separated by RLS status.

	Formal diagnosis of RLS	Symptoms of RLS no formal diagnosis	No RLS diagnosis/symptoms	P value
	(n=15)	(n=183)	(n=267)	
Age in years, mean (SD)	41 (7.4)	33.3 (8.5)	32.9 (8.2)	<0.01^1^
Hormonal medication, n (%)	4 (26.7)	73 (39.9)	97 (36.3)	0.5^2^
Smoker, n (%)	0/14 (0)	47/182 (25.8)	42/248 (16.9)	<0.01^2^
Alcohol consumption, n (%)	5/14 (35.7)	96/182 (52.7)	145/246 (58.9)	0.14^2^
Parous women, n (%)	12/14 (85.7)	98/182 (53.8)	119/246 (48.4)	0.02^2^
Anti-depressant use, n (%)	7 (46.7)	38 (20.8)	31 (11.6)	<0.01^2^

^1^ANOVA; ^2^Chi-squared

Of the 465 questionnaires analyzed, 71.8% (334) of the participants reported that they had symptoms associated with endometriosis, which we pre-defined as a response of ≥7 on VAS for any of the pain questions. However, only 99 (21.3%) out of those, self-reported receiving a formal surgical diagnosis of endometriosis. This gives a rate of formal-surgical diagnosis of endometriosis to be 29.6% (99/334) of the women suffering with endometriosis associated symptoms.

Women with a self-reported surgical diagnosis of endometriosis were older (P<0.01), more likely to use hormonal medications (P<0.01) and less likely to consume alcohol (P=0.05) than those without a prior diagnosis of endometriosis. Although there was an apparent trend for the women with a diagnosis of endometriosis to use more antidepressants, these differences did not reach statistical significance (P=0.06).

Among our study population, 42.6% (198) of patients had symptoms of RLS, but only 15 (3.2%) reported receiving a prior formal diagnosis of RLS. This gives a rate of formal RLS diagnosis to be 7.6% (15/198) among the women suffering with RLS related symptoms.

Women with a self-reported formal diagnosis of RLS were older (P<0.01), less likely to smoke (P<0.01), more likely to be parous (P=0.02) and more likely to use anti-depressants (P<0.01) than those without a prior diagnosis of RLS. The women with symptoms but no formal RLS diagnosis in post-hoc analysis were also significantly more likely to use antidepressants than those with no symptoms or diagnosis (**Table 2**).

Women with a self-reported surgical diagnosis of endometriosis were more likely to have a self-reported formal diagnosis of RLS (OR 4.82 95% CI (1.66, 14.02)) and symptoms suggestive of RLS (OR 2.13 95% CI (1.34, 3.39)) than those without a formal diagnosis of endometriosis (**Table 3**). Women with either a formal diagnosis or symptoms associated with endometriosis had a significantly increased risk of having either a formal diagnosis or symptoms suggestive of RLS (OR 2.49, 95% CI 1.30, 3.64).

**Table 3:** Outcomes.

	Formal diagnosis of RLS (n=15)	Symptoms of RLS no formal diagnosis (n=183)	No RLS diagnosis/symptoms(n=267)
Entire study population (n=465)	15 (3.2%)	183 (39.4%)	267 (57.4%)
Surgical endometriosis diagnosis (n=99)	7 (7.1%)	51 (51.5%)	41 (41.4%)
Endometriosis symptoms, no formal diagnosis (n=235)	6 (2.6%)	98 (41.7%)	131 (55.7%)
No diagnosis/symptoms of endometriosis (n=131)	2 (1.5%)	34 (26%)	95 (72.5%)

In women suffering with endometriosis-associated symptoms, the cumulative endometriosis-associated symptom scores demonstrated a modest positive correlation with RLS severity scores (r=0.42 95% CI 0.25 to 0.57).

## Discussion

This cross-sectional study suggests that there is a link between endometriosis and RLS. The data confirms that RLS symptoms are highly prevalent among non-pregnant woman during the reproductive years (gynecological population), and this is greater than the reported rates among the general population. Our results further suggest that a surgical diagnosis of endometriosis increased the likelihood of a formal diagnosis of RLS or women to be suffering with symptoms of RLS. Having either a formal diagnosis or symptoms associated with endometriosis in turn increased the likelihood of having a formal diagnosis or symptoms suggestive of RLS, and demonstrated a modest positive correlation between the severity of symptoms associated with endometriosis and RLS. This signifies the need to consider and treat both conditions simultaneously in these patients to improve their general wellbeing.

The prevalence of idiopathic RLS is reported to be between 1.9-4.6% of European adults (16, 23, 24) with a negative impact on their wellbeing (19). Those with early onset of symptoms (< 45 years) may have worsening of symptoms over time (19), therefore the observed high prevalence of symptoms suggestive of RLS among the non-pregnant women in the reproductive age (nearly 10-fold increase from the general population) is of concern. Almost 60% of those who recounted a surgical diagnosis of endometriosis, also reported experiencing an irresistible urge to move their legs due to an unpleasant sensation, a cardinal feature of RLS. However, a previous formal diagnosis of RLS, was only reported by 7.1% of women who had a diagnosis of endometriosis, suggesting a lack of awareness of this condition, thus, the majority of symptomatic women are not formally diagnosed.

The high prevalence of RLS symptoms in gynecological patients, and particularly in those who are suffering with symptoms associated with endometriosis or with a self-reported surgical diagnosis of endometriosis is therefore important for many reasons. Firstly, there may be common etiological factors between the two conditions that can be identified for novel therapeutic avenues. For example, iron deficiency is an identified causative factor for RLS (19) and RLS symptoms are exacerbated with iron deficiency (25). Albeit limited, there is evidence suggesting that women with endometriosis have significantly decreased values of hematocrit, hemoglobin concentrations and mean cell volume compared with age-matched controls (26). In a non-human primate (macaques) model of spontaneously occurring endometriosis, surgical diagnosis of endometriosis were associated with a significant increased rates of iron deficiency anemia, lower systemic iron stores, and decreased serum iron levels (26). Furthermore, women with endometriosis commonly complain of symptoms such as fatigue and malaise (27), which may be linked to or exacerbated by, iron deficiency. Therefore, evaluating common causal factors for RLS in women with endometriosis, (for example, conclusive assessment of the iron stores in women suffering with endometriosis associated symptoms) may unveil new therapeutic options of rectifying those abnormalities, in this context, iron deficiency.

Dopaminergic dysfunction is postulated to be involved (28) in the pathogenesis of RLS. RLS symptoms are typically worse at night and in the evening, resulting in insomnia, anxiety, depression, loss of energy, and disturbances in behavior, cognition and mood (14, 16, 29, 30).

The observed association between RLS and endometriosis should prompt clinicians to consider co-existence of symptoms, when managing women with either of these conditions. Both these chronic conditions require long term symptom management. Since the severity of the symptoms also seem to correlate positively with each other, clinicians seeing patients with one condition may need to seek for a diagnosis of the other and if found to co-exist, simultaneous treatment for both endometriosis and RLS associated symptoms is likely to improve overall wellbeing of the patients. The management pathway for RLS recommended by a task force for the European Restless Legs Syndrome Study Group (22) and NICE clinical knowledge summaries  (17) suggest lifestyle change advice and self-help measures could be sufficient in patients with mild RLS symptoms. These include good sleep hygiene, reducing caffeine and alcohol intake, smoking cessation and moderate regular exercise. Those patients with severe and disabling symptoms may significantly benefit from early detection and treatment (31). Non-pharmacological treatments (e.g. lifestyle change advice) could still be worthwhile in this patient group to decrease disease burden, but pharmacological treatment may be more appropriate. Interestingly, the first line pharmacological treatment for RLS may have a role in endometriosis management. For example, dopamine agonists may regress endometriotic lesions  (32, 33) and although not yet specifically tested in clinical trials, alpha-2-delta ligands such as gabapentin and pregabalin are commonly used in clinical practice to treat endometriosis associated pelvic pain (34, 35).

A major limitation of this study is not directly verifying the information from the self-completed questionnaires with the hospital records, and this needs to be considered when interpreting our results. For example, our study cohort included women with a self-reported surgical diagnosis of endometriosis and those with symptoms of endometriosis without a prior surgical diagnosis. This latter group would naturally include women who have either not had a diagnostic laparoscopy or those who have had endometriosis excluded by a diagnostic laparoscopy. Further appropriately designed studies in the future are warranted to explore differences in RLS symptoms in such potential subgroups of women. The calculation of a correlation coefficient between the sum of the RLS VAS and endometriosis VAS responses should be considered with caution due to the fact that although the IRLSSG Severity Rating Scale is validated for use on a continuous scale, the BSGE Pelvic Pain Questionnaire is not. The use of a single question ”Do you ever have the irresistible urge to move your legs due to an unpleasant sensation?”, will over-estimate true prevalence of symptoms suggestive of RLS, since we did not include all 5 essential diagnostic criteria according to IRLSSG (15, 16). Additionally, the VAS questions may potentially over-estimate the prevalence of symptoms associated with endometriosis due to subjective symptom exaggeration. Although our cohort included 465 fully completed questionnaires from women, our reported findings will need to be confirmed in a larger future cohort, which also allows investigation of the molecular mechanisms between the two conditions in order to draw definite conclusions.

An increased awareness of RLS in women with endometriosis and or those with associated symptoms, and screening women for RLS by their healthcare professionals may offer an opportunity to alleviate some common and synergistic symptoms (e.g. lack of sleep, low mood) and thus would be beneficial (36).

### Conclusion

This cross-sectional study builds on the current understanding of endometriosis and suggests that there is a link between symptoms of endometriosis and RLS.

Although the results of the study cannot be immediately applied to the wider population, the results are significant due to the substantial impact these two chronic conditions have upon quality of life, and improvements in knowledge can lead to a holistic approach to patient care. The results highlight a promising area of medicine, which could have a significant impact on care in these complex patients. Further research is needed to confirm or refute these findings, to encourage clinicians to actively seek for a diagnosis of RLS in the endometriosis population and if found to be present, offer treatments and lifestyle advice to relieve associated symptoms and improve the overall quality of life of thousands of women.

## Data Availability Statement

The original contributions presented in the study are included in the article/**Supplementary Material**. Further inquiries can be directed to the corresponding author.

## Ethics Statement

The studies involving human participants were reviewed and approved by North of Scotland Research Ethics Committee 2 (LREC: 17/NS/0070). Written informed consent for participation was not required for this study in accordance with the national legislation and the institutional requirements.

## Author Contributions

DH conceived the study and obtained ethical approval. AC and MB collected data and SL, MB, AC, and NT analyzed the data. MB, AC, NT, and DH interpreted the data and wrote the first draft of the manuscript. All authors contributed to the article and approved the submitted version.

## Funding

This work was funded by Wellbeing of Women fellowship grant (RTF510 NT and DH; RG2317 DH), NIHR ACL (NT), and the University of Liverpool (DH, NT, MB, and AC).

## Conflict of Interest

The authors declare that the research was conducted in the absence of any commercial or financial relationships that could be construed as a potential conflict of interest.
