# Supplementary material for: Premenopausal Women With a Diagnosis of Endometriosis Have a Significantly Higher Prevalence of a Diagnosis or Symptoms Suggestive of Restless Leg Syndrome: A Prospective Cross-Sectional Questionnaire Study
Source: Front Endocrinol (Lausanne). 2021 Mar 29;12:599306. doi: 10.3389/fendo.2021.599306 (PMC8040104; doi:10.3389/fendo.2021.599306)
Supplement: Supplementary file 1 [file DataSheet_1.pdf]

Supplementary Figure 1 – Study questionnaire.

<u>Questionnaire for investigating the incidence of restless legs in Gynaecological patients</u>

Please read the information leaflet provided. By completing this questionnaire, we assume that you agree to take part in this study and you have given your consent for the use of the anonymous information provided for research purposes. This questionnaire is anonymous and confidential, the information will only be used for this project and will not be shared with anyone who isn't involved in the research.

Thank you for your time.

Please circle the most appropriate answer that suits you best or write on the line provided.

1. How old are you currently? _______
2. Are you currently pregnant? **YES** **NO**
3. Please could you provide a list of medications you are **currently taking**:

____

____

____

____

____

4. Are you currently taking any of the following treatments?
   - ☐ **Mirena IUS (hormonal coil). YES NO** If yes, for how long? ____________________
   - ☐ **GnRH analogues (e.g. Prostap).** If so, for how long? ________________________
   - ☐ **HRT.** If so, for how long? _________________________
   - ☐ **Combined oral contraceptive pill (COCP). YES NO** If yes, for how long? _________________________
   - ☐ **Depo-provera injection. YES NO** If yes, for how long? _________________________
   - ☐ **Contraceptive Implant. YES NO** If yes, for how long? _________________________
   - ☐ **Mini-pill. YES NO** If yes, for how long? __________________________________
   - ☐ **Painkillers YES NO** If yes, for how long? __________________________________
5. Do you have regular periods? **YES NO** if yes, are they **Heavy Normal Light**
6. Have you been diagnosed with endometriosis? **YES NO** If yes, when were you diagnosed? ____
7. Did you have surgery to confirm that you have endometriosis? YES NO If yes, when _______________
8. Do you smoke cigarettes? **YES NO** If yes, how many? ________/day
9. Do you drink alcohol? **YES NO** If yes, how much? _____________units/week
10. Have you had any children? **YES NO** If yes, how many? _____________

During your **normal monthly menstrual cycle,** which of the following symptoms do you experience now?

Please circle **YES**, **NO** or **not applicable (N/A)** to show whether you experience these symptom during a normal cycle or not, and then if you have experienced this symptom, circle a score from 1 to 10 to indicate how slight or severe it usually is.

1. **Pain before periods (Premenstrual pain).** Experienced **YES** **NO** **N/A**

**1 Experienced slightly**	**2**	**3**	**4**	**5**	**6**	**7**	**8**	**9**	**10 Experienced severely**

2. **Pain during periods (Menstrual pain).** Experienced **YES** **NO** **N/A**

**1 Experienced slightly**	**2**	**3**	**4**	**5**	**6**	**7**	**8**	**9**	**10 Experienced severely**

3. **Pain throughout the month (Non-cyclical pelvic pain)** Experienced **YES** **NO** **N/A**

**1 Experienced slightly**	**2**	**3**	**4**	**5**	**6**	**7**	**8**	**9**	**10 Experienced severely**

4. **Pain during sexual intercourse.** Experienced **YES** **NO** **N/A**

**1 Experienced slightly**	**2**	**3**	**4**	**5**	**6**	**7**	**8**	**9**	**10 Experienced severely**

5. **Pain opening bowels during period.** Experienced **YES** **NO** **N/A**

**1 Experienced slightly**	**2**	**3**	**4**	**5**	**6**	**7**	**8**	**9**	**10 Experienced severely**

6. **Pain opening bowels at other times (not during period)** Experienced **YES** **NO** **N/A**

**1 Experienced slightly**	**2**	**3**	**4**	**5**	**6**	**7**	**8**	**9**	**10 Experienced severely**

7. **Lower back pain.** Experienced **YES** **NO** **N/A**

**1 Experienced slightly**	**2**	**3**	**4**	**5**	**6**	**7**	**8**	**9**	**10 Experienced severely**

8. **Bladder pain.** Experienced **YES** **NO** **N/A**

**1 Experienced slightly**	**2**	**3**	**4**	**5**	**6**	**7**	**8**	**9**	**10 Experienced severely**

1. Do you ever have the irresistible urge to move your legs due to an unpleasant sensation? **YES NO**

If you answered yes, please continue.

2. Have you ever been diagnosed with restless leg syndrome? **YES** **NO**
3. Have you ever felt an urge in the arms (upper limbs)? **YES** **NO**
4. Is it worse at night? **YES** **NO**
5. Is it worse at rest? **YES** **NO**

In response to the following questions, please circle the most appropriate score for you.

6. Overall, how would you rate the discomfort in your limbs?

**0 None**	**1 Mild**	**2 Moderate**	**3 Severe**	**4 Very severe**

7. Overall, how would you rate the need to move around because of your symptoms?

**0 None**	**1 Mild**	**2 Moderate**	**3 Severe**	**4 Very severe**

8. Overall, How much relief of your symptoms do you get from moving around?

**0 No symptoms so doesn’t apply.**	**1 Complete or almost complete relief.**	**2 Moderate relief**	**3 Slight relief**	**4 No relief**

9. Overall, how severe is your sleep disturbance from your symptoms?

**0 None**	**1 Mild**	**2 Moderate**	**3 Severe**	**4 Very severe**

10. How severe is your tiredness or sleepiness from your symptoms?

**0 None**	**1 Mild**	**2 Moderate**	**3 Severe**	**4 Very severe**

11. Overall, how severe are your symptoms as a whole?

**0**<br>**None**	**1**<br>**Mild**	**2**<br>**Moderate**	**3**<br>**Severe**	**4**<br>**Very severe**

12. How often do you get these symptoms?

**0**<br>**None**	**1**<br>**1 day per week or less.**	**2**<br>**2-3 days per week.**	**3**<br>**4-5 days per week.**	**4**<br>**6-7 days per week.**

13. When you have these symptoms, how severe are they on an average day?

**0**<br>**None**	**1**<br>**<1 hour per day.**	**2**<br>**1-3 hours per day.**	**3**<br>**3-8 hours per day.**	**4**<br>**8 hours or more per day.**

14. Overall, how severe is the impact of your symptoms on your ability to carry out your daily activities, for example carrying out a satisfactory family, home, social, school or work life?

**0**<br>**None**	**1**<br>**Mild**	**2**<br>**Moderate**	**3**<br>**Severe**	**4**<br>**Very severe**

15. How severe is your mood disturbance from your symptoms, for example angry, depressed, sad, anxious or irritable?

**0**<br>**None**	**1**<br>**Mild**	**2**<br>**Moderate**	**3**<br>**Severe**	**4**<br>**Very severe**

Thank you.
